# Oxidative stress generated DNA damage by 6PPD and other tyre additives in A549 human lung epithelial cells

**DOI:** 10.1038/s41598-025-19232-y

**Published:** 2025-10-13

**Authors:** Samuel Hyman, Rea Bilić, Annie Jensen, Siriel Saladin, Yurii Tsybrii, Oleksii Nosko, David Topping, Adam Boies, Chiara Giorio, Martin Roursgaard, Peter Møller

**Affiliations:** 1 Department of Earth and Environmental Science, Centre for Atmospheric Science, School of Natural Sciences, The University of Manchester, Manchester, UK; 2 Section of Environmental Health, Department of Public Health, University of Copenhagen, Copenhagen, Denmark; 3 Yusuf Hamied Department of Chemistry, University of Cambridge, Cambridge, UK; 4 Faculty of Mechanical Engineering and Ship Technology, Gdansk University of Technology, Gdansk, Poland; 5 Department of Engineering, University of Cambridge, Cambridge, UK

**Keywords:** 6PPD, 6PPDQ, Benzothiazole, Oxidative stress, Glutathione, DNA damage, Nucleus, Environmental impact, DNA damage and repair

## Abstract

Tyre additives such as *p*-phenylenediamines (PPDs) and benzothiazoles (BTs) are ubiquitous in the environment. They have been frequently detected in urban air and have been detected in the human body. However, few studies have examined the toxicological effects in human cells. In this study we perform cytotoxicity, oxidative stress and DNA damage assays on A549 human alveolar lung cells with *N*-(1,3-dimethylbutyl)-*N*′-phenyl-*p*-phenylenediamine (6PPD), *N*-(1,3-dimethylbutyl)-*N*′-phenyl-*p*-phenylenediamine-quinone (6PPD-Q), diphenyl-*p*-phenylenediamine (DPPD), 1,3-benzothiazole (BTZ) and 2-mercaptobenzothiazole (MBT). It was found that all additives were able to cause glutathione (GSH) depletion and induce DNA strand breaks after 24 h exposure in a concentration-dependent manner. The presence of *N*-acetyl-L-cysteine (NAC), a GSH precursor, mitigated both GSH depletion and DNA damage from 6PPD. Although the tested concentrations of tyre additives exceeded typical levels reported for ambient air, these additives have been detected in wastewater, road runoff and road dust. Therefore, human exposure can occur through multiple routes, including inhalation, ingestion, and dermal absorption, ultimately reaching alveolar cells either directly via the lungs or indirectly through the bloodstream.

## Introduction

To improve the performance, safety and durability of rubber, numerous chemical additives are incorporated during the manufacturing process of tyres. Substituted *p*-phenylenediamine antioxidants (PPDs) such as *N*-(1,3-dimethylbutyl)-*N*′-phenyl-*p*-phenylenediamine (6PPD) and diphenyl-*p*-phenylenediamine (DPPD) are used to safeguard against environmental stressors as UV light, heat and ozone. Benzothiazoles (BTs) such as 1,3-benzothiazole (BTZ) and 2-mercaptobenzothiazole (MBT) are used as vulcanization accelerators to enhance mechanical strength and abrasion resistance. These additives may undergo further reactions to form breakdown products; for example, 6PPD reacts with ozone to produce 6PPD-quinone (6PPD-Q).

Tyre additives are emitted into the environment via tyre wear particles, which are abraded from the tyre at the interface with the road during steady driving, accelerating, and braking. Occupational exposure may also represent a significant source of human contact with these additives, such as in the case of certain factory workers^1–5^. Shear and friction forces tend to produce non-airborne particles with relatively large aerodynamic diameter (> 10 μm), whilst friction-related heat was observed to generate smaller particles (< 0.1 μm), which are capable of being inhaled into the lungs. The former particles dominate mass distribution^6^ while the latter dominate by number^7^. The emission factors of directly emitted airborne tyre particles with diameters between 0.1 and 10 µm by mass (not necessarily number) appear lower than anticipated in the years 1995–2025^8^. Nevertheless, big (non-airborne) tyre particles can degrade over time leading to smaller particles which contribute to road dust and eventually could become airborne via resuspension. The absolute magnitude of this indirect emission pathway is unknown. The concerns, however, are increasing because of a global trend toward heavier vehicles such as electric cars that are associated with increased weight as well as torque. Both factors have been observed to drive the mass of emitted tyre wear particles per driven distance^9^. The composition of tyres is largely undisclosed, making tyres a black box to academic researchers. Additionally, there are currently no policies in place to regulate tyre-related pollutants such as 6PPD and its transformation products, including 6PPD-Q^10^.

The presence of 6PPD, 6PPD-Q, and DPPD in particulate matter with aerodynamic diameter ≤ 2.5 μm (PM_2.5_) or ≤ 10 μm (PM_10_) was reported for ambient air in the order of pg/m^3^^11–14^, with similar findings for BTs^15^. Additionally, these additives have been detected in wastewater, road runoff and road dust^16–18^. This is relevant as human exposure can occur through multiple routes, including inhalation, ingestion, and dermal absorption, ultimately reaching alveolar cells either directly via the lungs or indirectly through the bloodstream. Evidence also suggests that 6PPD-Q may compromise intestinal barrier integrity at environmentally relevant or lower levels, potentially increasing the absorption of contaminants via ingestion^19^. 6PPD and/or 6PPD-Q have been detected in human urine, blood and cerebrospinal fluid^20–22^. 6PPD-Q has also been shown to reach higher levels in the lungs compared to other organs tested in a mouse model after repeated injections, possibly due to its distribution pattern or the high blood supply of the lungs^23^. BTZ and MBT have been detected in human exhaled breath, urine and adipose tissue^24–26^.

Despite their ubiquitous nature, toxicological data on 6PPD, 6PPD-Q, and DPPD remains limited with notably few studies looking at effects on a molecular level. Recent literature has suggested that these compounds can induce oxidative stress that may contribute to toxic outcomes such as mitochondrial stress, DNA adduct formation and disrupted lipid metabolism^21,22,27,28^. Similarly, research on BTZ and MBT is lacking, yet there is evidence that they can cause oxidative stress^29–31^. This study provides novel insights into the potential of five tyre additives/breakdown products (6PPD, 6PPD-Q, DPPD, BTZ and MBT) to contribute to oxidative stress and cause DNA damage in human lung epithelial A549 cells.

## Methods

### Exposure of A549 cells with tyre additives.

#### Chemicals

**Fig. 1:**
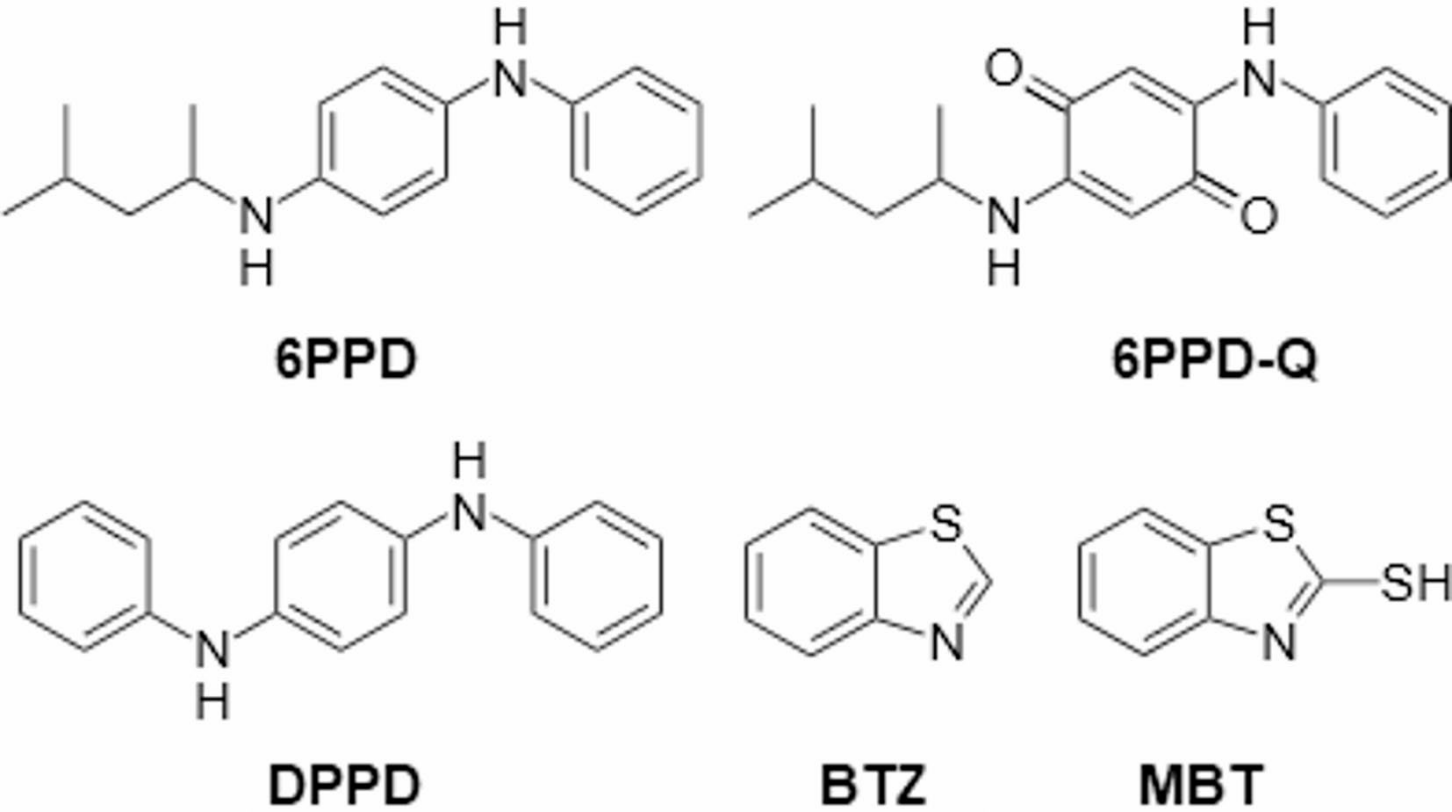
Chemical structures of the investigated tyre additives.

Buthionine sulfoximine (BSO) (CAS No. 5072-26-4), diethyl maleate (DEM) (CAS No. 141-05-9), *N*-acetyl-L-cysteine (NAC) (CAS No. 616-91-1), hydrogen peroxide (H_2_O_2_) (CAS No. 7722-84-1), Triton™ X-100 (CAS No. 9036-19-5), 6PPD (CAS No. 793-24-8), DPPD (CAS No. 74-31-7), BTZ (CAS No. 95-16-9) and MBT (CAS No. 149-30-4) were purchased as pure chemicals from Sigma-Aldrich. 6PPD-Q (CAS No. 2754428-18-5) was purchased as a pure chemical from LGC Standards. The concentrations in this study were selected to assess acute effects and were not based on environmentally relevant levels. Some compounds exhibited limited solubility at these concentrations, which prevented testing at higher levels. The selected concentrations also fell within the range used in previous studies, which allowed comparison with existing literature. Figure 1 shows the chemical structures of the tyre additives.

#### Cell culture and exposure

A549 cells (American Type Culture Collection, Manassas, Virginia, USA) were cultured in F12 Ham Nutrient mix supplemented with 10% foetal bovine serum (FBS), 1% L-glutamine, and 1% penicillin/streptomycin (all from Gibco). Cells were incubated at 37 °C and 5% CO_2_. A549 cells are useful for detection of oxidative stress and some types of DNA damage (i.e. DNA strand breaks and oxidatively damaged DNA, measured by the comet assay), although they may not be the optimal cell line for detection of certain other types of genotoxicity (e.g. chromosome damage or mutations) or non-genotoxic endpoints (e.g. inflammation). Prior to exposure, cells were cultured in cell medium for 24 h before being exposed to tyre additives or positive controls for either 3 or 24 h. These time points were chosen as optimal durations of exposure for the investigated endpoints; they do not necessarily reflect a realistic or relevant duration of exposure in humans. Negative control groups consisted of untreated cells in cell culture media. All exposures were repeated in at least three independent experiments conducted on separate days.

### Cytotoxicity (WST-1 and LDH assays)

Cytotoxicity was evaluated using a combination of the intracellular conversion of water-soluble tetrazolium 1 (WST-1) to formazan by dehydrogenase enzymes and the measurement of lactate dehydrogenase (LDH) activity in the cell medium, which serves as an indicator of cell membrane integrity^32^. 50,000 cells per well were seeded into transparent 96-well plates 24 h before being exposed for an additional 24 h to tyre additives or 1% Triton™ X-100 as a positive control. Following exposure, 100 μL of supernatant was transferred to a new transparent 96-well plate, and 100 μL of LDH working solution (Lactate Dehydrogenase Activity, Roche Diagnostics) was added to each well. The plate was covered with aluminium foil and left at room temperature for 15 min. LDH activity was then quantified by spectrophotometric analysis, measuring absorbance at 500 nm with 630 nm as the reference wavelength (Multiskan FC Microplate Photometer, Thermo Fisher). To assess cellular dehydrogenase activity, the culture medium was completely removed from the wells, and 100 μL of WST-1 reagent (10%) prepared in fresh culture medium was added. Cells were incubated at 37 °C for 1 h before absorbance was measured at 500 nm, with 630 nm as the reference wavelength, using a Multiskan™ FC Microplate Photometer (Thermo Fisher Scientific).

### Intracellular ROS production

Intracellular reactive oxygen species (ROS) production was assessed using the 2′,7′-dihydrofluorescein diacetate (DCFH-DA) assay^32^. 50,000 cells per well were seeded in black 96-well plates 24 h prior to exposure. On the day of the experiment, the cell culture medium was replaced with Hank’s Balanced Salt Solution (HBSS; Gibco) containing 10 µM DCFH-DA probe. After a 15-min incubation period at 37 °C, cells were washed once with 200 μL HBSS to remove any excess extracellular probe. The cells were then exposed to tyre additives in HBSS for 3 h. H_2_O_2_ at concentrations ranging from 100 to 500 μM served as a positive control. The fluorescence signal was measured at an excitation wavelength of 490 nm and an emission wavelength of 520 nm, using a Multiskan™ FC Microplate Photometer (Thermo Fisher Scientific).

### Intracellular glutathione level

Intracellular glutathione (GSH) levels were assessed as described previously^32^. 50,000 cells per well were seeded in black 96-well plates 24 h before exposure. The cells were exposed for either 3 or 24 h in complete cell culture medium to tyre additives or positive controls (which were DEM alone or in combination with BSO). DEM concentrations ranged from 0 to 1 mM, while certain wells also received 50 µM BSO (final concentration) by adding 2.5 µL of a 4 mM BSO stock solution to 200 µL of medium per well. In a separate experiment, the cell culture medium was supplemented with 10 mM NAC at the start of the 3-h or 24-h exposure period. Following the exposure, the supernatant was removed, and wells were washed with 200 µL phosphate-buffered saline (PBS). A ThioGlo-1 stock solution (Covalent Technologies, Inc., Walnut Creek, CA, USA) was diluted to a 10 μM working concentration for further dilutions as needed. 100 μL of ThioGlo-1 working solution was added to each well. Plates were incubated at room temperature for approximately 5 min before fluorescence was measured (excitation: 355 nm; emission: 460 nm), using a Multiskan™ FC Microplate Photometer (Thermo Fisher Scientific). Intracellular GSH levels were quantified using a GSH standard curve ranging from 0.125 to 16 μM and reported as nmol/10^6^ plated cells.

### DNA strand breaks (comet assay)

Cells were seeded at a density of 250,000 cells/well in transparent 24-well plates 24 h before exposure. Following this incubation, the culture medium was removed, and cells were exposed to tyre additives for 24 h (main experiment). In a separate experiment, cell culture medium with 10 mM NAC was added at the start of the 24 h exposure period. At the end of the exposure, single-cell suspensions were prepared by discarding the culture medium, washing cells with 1 mL PBS, and trypsinization with 150 μL trypsin per well. After a 5-min incubation at 37 °C with 5% CO_2_, 350 µL of cell culture medium was added to terminate the enzymatic activity of the trypsin.

A 100 µL aliquot of the resulting cell suspension was mixed with 600 µL of 0.75% agarose, and 120 µL of this mixture was applied to GelBond films in duplicate. Cells were lysed at 4 °C for 1 h in lysis solution (2.5 M NaCl, 0.1 M Na₂EDTA, 10 mM Tris, 1% Triton™ X-100, pH 10), briefly rinsed in PBS, and placed in electrophoresis buffer (1 mM Na₂EDTA and 300 mM NaOH, with recycling of the solution) for 40 min at 4 °C. Electrophoresis was then performed for 25 min at 300 mA and 20 V (0.83 V/cm; voltage measured across the electrophoresis platform was 12.8 V or 0.53 V/cm)^33^. Following electrophoresis, GelBond films were neutralized in 0.4 M Tris buffer for 15 min and left overnight in 96% ethanol. After drying, the films were cut, and DNA was stained with YOYO-1 (ThermoFisher Scientific). Comet assay scoring was conducted under blinded conditions using a fluorescence microscope at 40 × magnification, with 100 cells per gel analysed using a five-class scoring system (total of 200 comets; scoring range: 0–100 arbitrary units (a.u.))^34^. The visual score in arbitrary units was converted to lesions per 10^6^ base pairs using investigator-specific calibration curves based on gamma radiation^35^. Two investigators performed the experiments: Investigator #1 analysed 6PPD, DPPD, BTZ, and MBT, while Investigator #2 examined 6PPD, 6PPD-Q, BTZ, and MBT. Calibration curves were as follows: Investigator #1-1.6, 14.1, 47.1, and 66.8 a.u. for THP-1 cells exposed to 0, 2.5, 5, and 7.5 Gy (no scoring of the 10 Gy sample); 0.0206 lesions per 10⁶ base pairs per 1 a.u. in 0–100 a.u. scale. Investigator #2-3.1, 37.4, 62.5, 77.0, and 89.0 a.u. for THP-1 cells exposed to 0, 2.5, 5, 7.5, and 10 Gy; 0.0233 lesions per 10⁶ base pairs per 1 a.u in 0–100 a.u. scale. The calibrated results from both investigators were combined.

No general threshold for cytotoxicity was found at which the test results of the comet assay were flawed. A 25% increase in cytotoxicity relative to the concurrent negative control is generally considered a reasonable threshold to avoid false positive comet assay results, although even cytotoxicity levels up to 50% does not seem to affect background levels DNA strand break levels^36^.

### Statistical analysis

The results have been analysed as concentration–response relationship (non-parametric trend test) and ANOVA. Adjustments for inter-experiment variability were made for comet assay and GSH data, with Sidak post-hoc test for differences between exposure groups. For experiments involving two factors (e.g., tyre additives and NAC, or DEM and BSO treatment), a full factorial ANOVA was applied. Results are expressed as the mean and standard error of the mean (SEM). The net effects of biomarkers, along with their 95% confidence intervals (95% CI), are reported to give an impression of the effect sizes and experimental variation. Statistical analyses were performed using Stata 15 (StataCorp LLC, College Station, TX, USA).

## Results

### Cytotoxicity

**Fig. 2:**
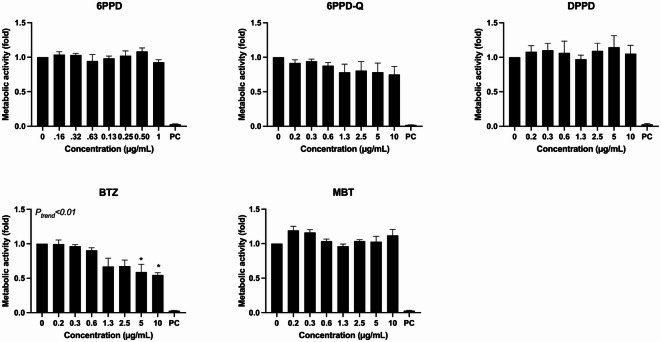
Effect of tyre additives on cellular metabolic activity (i.e. conversion of WST-1 to formazan by cellular dehydrogenases) at 24 h exposure in A549 cells. Bars and error bars are mean and SEM, respectively, of at least three independent experiments. *P < 0.05 compared to control group. *P* values for trend tests are shown on the graphs to indicate concentration–response relationships.

Figure 2 shows the effect of exposure on cellular metabolic activity (WST-1 assay) after 24 h. The exposure to BTZ was associated with concentration-dependent reduction of metabolic activity (*P* < 0.01, trend test). The highest BTZ concentration was associated with 45% reduction (95% CI 27%, 67%) of the metabolic activity. A non-linear dependency of the response is observed in the 24-h LDH assay, as well as in the 3-h ROS and GSH assays. While this may be attributed to variability across the three experimental repeats, further investigation could help better understand the underlying factors.

**Fig. 3:**
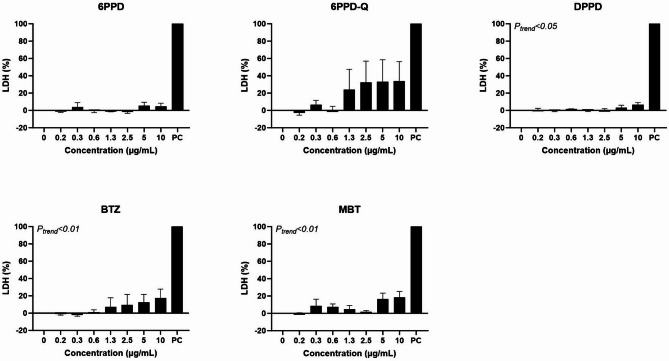
Effect of tyre additives on cell membrane permeability (i.e. lactate dehydrogenase activity in cell culture medium) at 24 h exposure in A549 cells. Bars and error bars are mean and SEM, respectively, of at least three independent experiments. *P < 0.05 compared to control group. *P* values for trend tests are shown on the graphs to indicate concentration–response relationships.

Figure 3 shows the effect of exposure on cell membrane permeability (LDH activity in cell culture medium) after 24 h. The exposure to DPPD, BTZ and MBT were associated with concentration-dependent increase of LDH activity in cell culture medium (*P* < 0.05 for DPPD and *P* < 0.01 for BTZ and MBT, trend test). The highest concentration was associated with 6.5% (95% CI 1.4%, 12%), 18% (95% CI 4%, 33%) and 18% (− 5%, 40%) increased LDH activity in cell culture medium of cells exposed to DPPD, BTZ and MBT, respectively.

### Intracellular ROS production

**Fig. 4:**
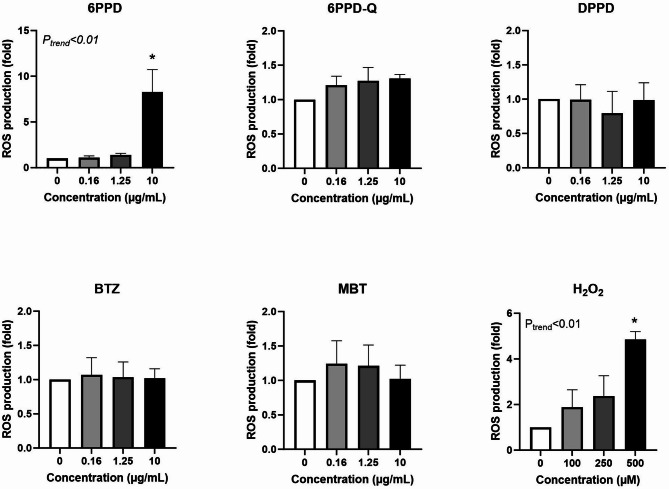
Effect of tyre additives on intracellular reactive oxygen species (ROS) production at three hours exposure in A549 cells. Bars and error bars are mean and SEM, respectively, of at least three independent experiments. *P < 0.05 compared to control group. *P* values for trend tests are shown on the graphs to indicate concentration–response relationships.

Figure 4 shows the effect of exposure on intracellular ROS production after 3 h. 6PPD showed a large increase in intracellular ROS production at 10 μg/mL, whereas the remaining tyre additives did not cause an effect. The positive control (H_2_O_2_) increased the intracellular ROS production 4.9-fold (95% CI 2.9, 6.8-fold).

### Intracellular glutathione levels

**Fig. 5:**
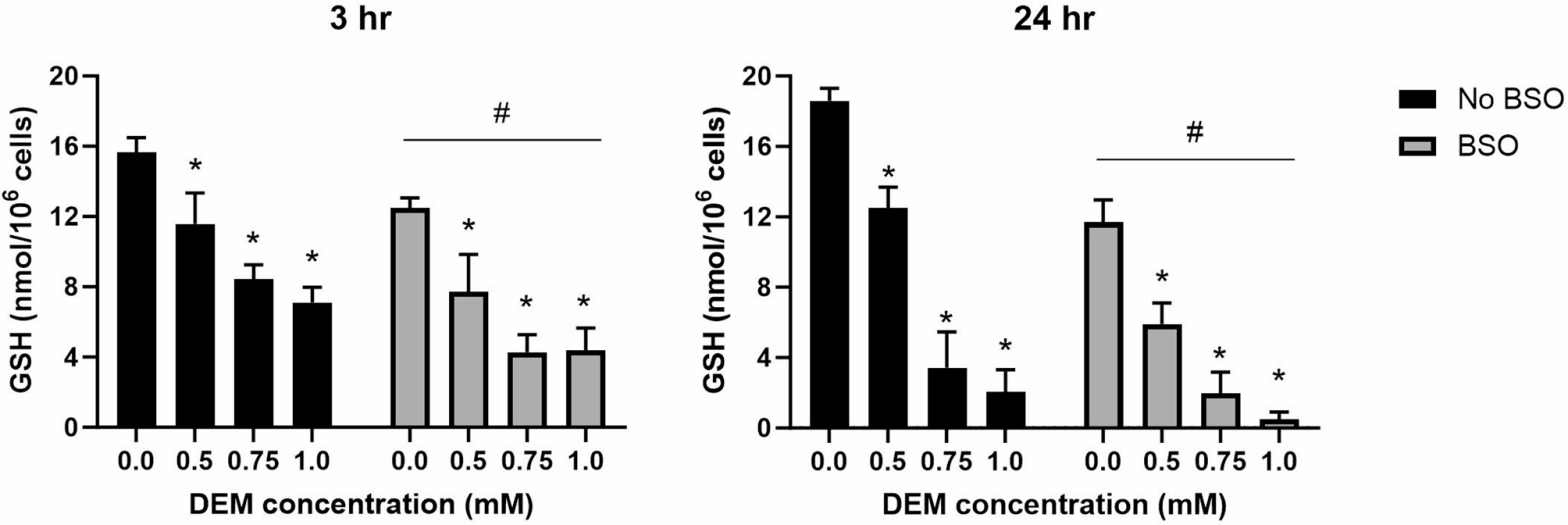
Effect of DEM and BSO treatment on GSH levels in A549 cells. Some wells were additionally treated with 50 µM BSO (final concentration) by adding 2.5 µL of 4 mM BSO stock to 200 µL of solution in each well. Bars and error bars are mean and SEM, respectively, of three independent experiments. *P < 0.05 compared to control group without DEM exposure (i.e. single-factor effect of BSO). ^#^*P* < 0.05 compared to cells without exposure to BSO (i.e. single-factor effect of DEM).

Glutathione status in the exposure periods was first assessed by exposures to positive controls that cause glutathione depletion by degradation (i.e. DEM) or blockage of glutathione synthesis (i.e. BSO) (Fig. 5). After 3 h exposure, there were single-factor effects of DEM (*P* < 0.001) and BSO (*P* < 0.01), whereas there was no interaction between DEM and BSO. The net effect of BSO treatment was − 3.5 nmol/10^6^ cells (95% CI − 5.4, − 1.6 nmol/10^6^ cells). For DEM exposure, there was a concentration-dependent effect (e.g. the lowest concentration caused a decrease of − 4.4 nmol/10^6^ cells (95% CI − 7.2, − 1.8 nmol/10^6^ cells). Similar effects were seen after 24 h exposure (i.e. single-factor effects of DEM (*P* < 0.001) and BSO (*P* < 0.001), and no interaction between DEM and BSO). The single-factor effect of BSO was − 4.2 nmol/10^6^ cells (95% CI − 6.0, − 2.2 nmol/10^6^ cells). For DEM exposure, the lowest concentration was associated with a decrease of − 6.0 nmol/10^6^ cells (95% CI − 8.6, − 3.2 nmol/10^6^ cells). Collectively, experiments on DEM and BSO positive controls show a somewhat stronger effect after 24 h exposure as compared to 3 h.

**Fig. 6:**
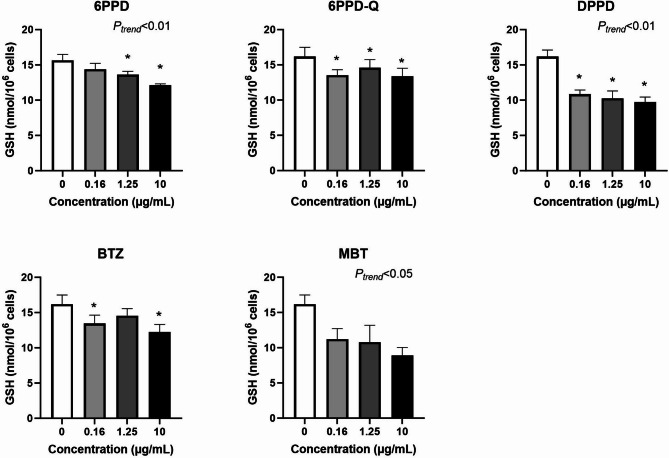
Effect of tyre additives on GSH levels at three hours exposure in A549 cells. Bars and error bars are mean and SEM, respectively, of at least three independent experiments. *P < 0.05 compared to control group. *P* values for trend tests are shown on the graphs to indicate concentration–response relationships.

**Fig. 7:**
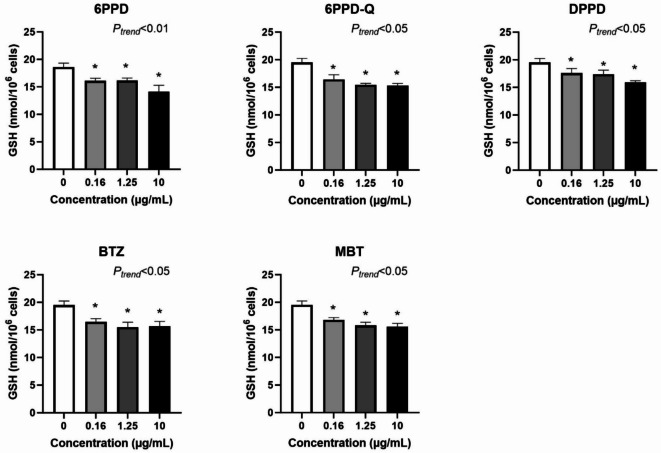
Effect of tyre additives on GSH levels at 24 h exposure in A549 cells. Bars and error bars are mean and SEM, respectively, of at least three independent experiments. *P < 0.05 compared to control group. *P* values for trend tests are shown on the graphs to indicate concentration–response relationships.

Figure 6 shows the effect of tyre additives on GSH levels after 3 h exposure. All additives showed an effect, as assessed by either trend test (6PPD, DPPD, and MBT) or ANOVA (6PPD-Q and BTZ). 6PPD was the additive with most robust association as demonstrated by statistical significance in both trend test and ANOVA. However, the net effect was not substantially different between 3 and 24 h as demonstrated by results on 6PPD (i.e. − 3.6 nmol/10^6^ cells (95% CI − 5.4, − 1.8 nmol/10^6^ cells) and − 4.4 nmol/10^6^ cells (95% CI − 6.4, − 2.6 nmol/10^6^ cells)). The effect of all tyre additives on GSH depletion was more robust at 24 h exposure, with statistically significant effect in both trend test and ANOVA (Fig. 7).

### DNA damage

**Fig. 8:**
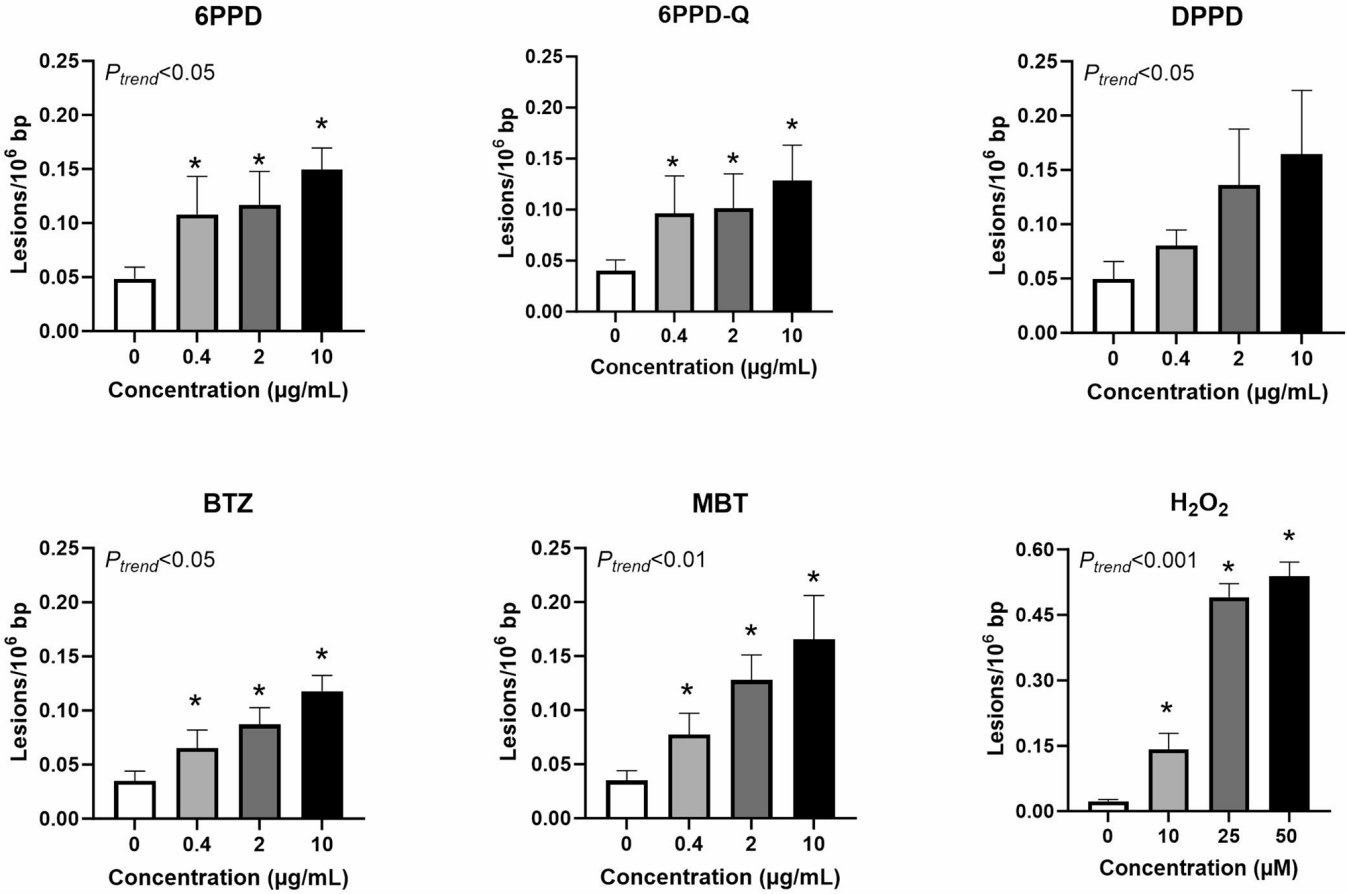
Levels of DNA strand breaks in A549 cells at 24 h exposure to tyre additives. Bars and error bars are mean and SEM, respectively, of at least five independent experiments. *P < 0.05 compared to control group. *P* values for trend tests are shown on the graphs to indicate concentration–response relationships.

Levels of DNA strand breaks were assessed by the comet assay after 24 h exposure (Fig. 8). Genotoxicity by exposure to 6PPD, BTZ and MBT was statistically significant on both trend test and ANOVA (*P* < 0.05), whereas 6PPD-Q (*P* < 0.05, ANOVA) and DPPD (*P* < 0.05, trend test) only showed statistically significant increase in one type of test.

### Effect of NAC supplementation on intracellular GSH levels and DNA damage

**Fig. 9:**
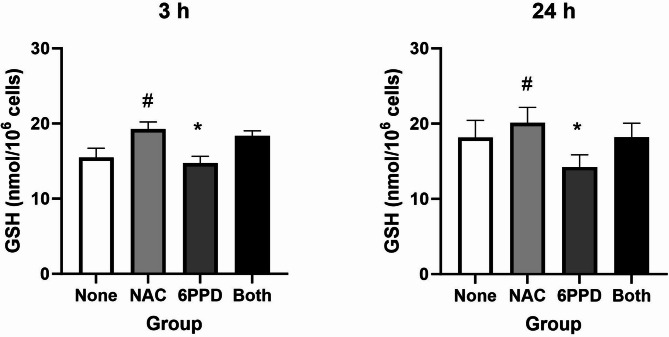
Effect of NAC supplementation on 6PPD-induced glutathione (GSH) depletion at 24 h exposure in A549 cells. Concentration of 6PPD used was 10 µg/mL. Bars and error bars are mean and SEM, respectively, of 3 independent experiments. *P < 0.05 for single-factor effect of 6PPD (i.e. decreased GSH concentration in cells exposed to 6PPD). #*P* < 0.05 for single-factor effect of NAC supplementation (i.e. increased glutathione in cells supplemented with NAC).

**Fig. 10:**
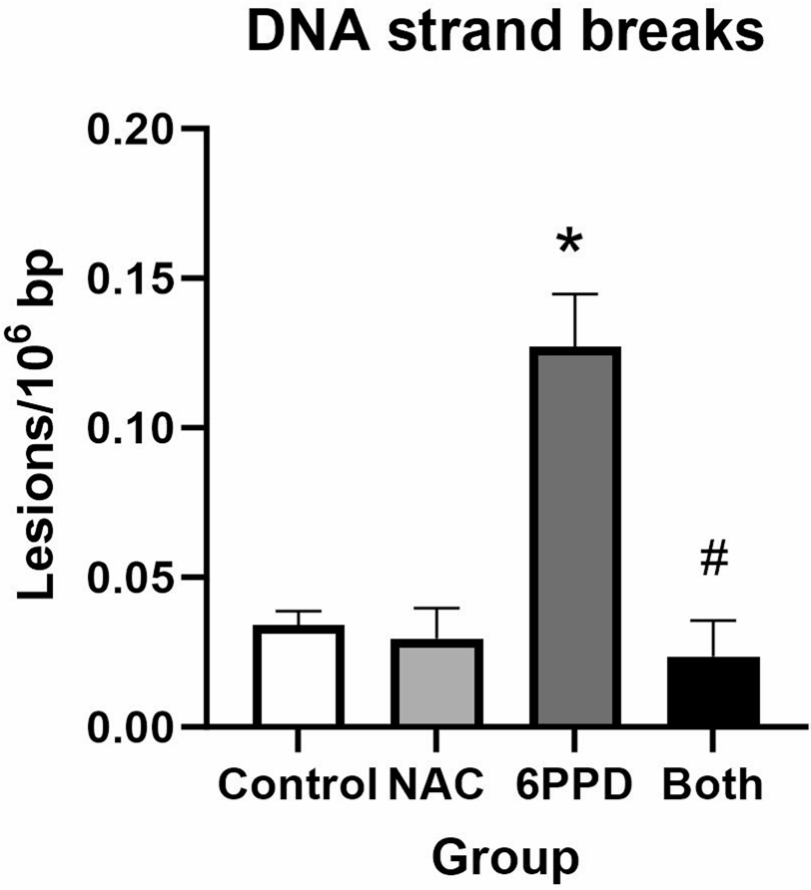
Effect of NAC supplementation on 6PPD-induced DNA damage at 24 h exposure in A549 cells. Concentration of 6PPD used was 10 µg/mL. Bars and error bars are mean and SEM, respectively, of 3 independent experiments. *P < 0.05 for single-factor effect of 6PPD (i.e. increased level of DNA strand breaks in cells exposed to 6PPD). ^#^*P* < 0.01 for interaction between 6PPD and NAC (i.e. NAC inhibits the genotoxic effect of 6PPD).

In order to assess the relationship between tyre additive exposure, GSH depletion and DNA damage, we supplemented A549 cell cultures with NAC 10 mM at the start of the 24 h exposure period (Fig. 9). In these experiments, we have used 6PPD because it is the additive that has been most widely investigated and it caused GSH depletion at both 3 and 24 h. The results show that depletion of GSH by 6PPD is blunted by administration of NAC (i.e. statistically non-significant difference between controls and NAC/6PPD co-exposure at 3 and 24 h). The effect on GSH depletion of 6PPD exposure without NAC was weaker at 3 h (− 0.8 nmol/10^6^ cells, 95% CI − 1.6, − 0.1 nmol/10^6^ cells) as compared to 24 h (− 5.0 nmol/10^6^ cells, 95% CI − 4.6. − 1.2 nmol/10^6^ cells). A slightly lower increase of GSH concentration without 6PPD was also seen for the NAC supplementation at 3 h (3.8 nmol/10^6^ cells, 95% CI 3.0, 4.4) as compared to 24 h (3.0 nmol/10^6^ cells, 95% CI 1.4, 4.6 nmol/10^6^ cells). Supplementation with NAC 10 mM in 6PPD-exposed A549 cells inhibited the genotoxic effect (*P* < 0.01 for interaction between 6PPD and NAC exposure on levels of DNA strand breaks, Fig. 10).

## Discussion

This study investigated the toxicological effects of five tyre additives or breakdown products: PPDs (6PPD, 6PPD-Q, DPPD), and BTs (BTZ and MBT) on human alveolar A549 cells. We observed that these additives caused GSH depletion and induce DNA strand breaks after 24 h exposure at all concentrations tested (0.16–10 μg/mL) in a concentration-dependent manner. Adding the GSH precursor and antioxidant, NAC, at the exposure stage with 6PPD mitigated both reduction in GSH levels and DNA damage, implying a causal relationship between oxidative stress and the DNA damage observed in this study.

### PPDs

Our finding that 6PPD, 6PPD-Q and DPPD did not affect cell cytotoxicity up to a concentration of 10 μg/mL matches previous literature with human alveolar A549 cells. However, using a wider concentration range up to 50 μg/mL, Chen et al.^37^ demonstrated that 6PPD significantly impacted cell viability in both the MTS (i.e. assay similar to the WST-1 assay) and ATP detection assay and induced IL-1B release in a concentration-dependent manner across several human cells (A549, THP-1 and BEAS-2B). Chen et al. did not observe these effects with 6PPD-Q. In human myeloid HL-60 cells, 6PPD was found to reduce cell viability at concentrations of 2.7 µg/mL or higher^38^. In contrast, 6PPD-Q, and not 6PPD, decreased cell viability in human liver cells (L02 and HepG2) at concentrations below 1 µg/mL when assessed with the MTT assay^39^. In human kidney cells, both 6PPD and 6PPD-Q reduced cell viability in a CCK-8 assay at similar concentrations to our study, with 6PPD-Q showing a stronger effect^40^. In another study, the WST-8 assay, which is particularly sensitive to cytosolic and extracellular enzyme activities, with concentrations below 0.1 µg/mL, 6PPD, 6PPD-Q, and DPPD reduced cell viability in HepG2 cells^41^. In addition, studies in other species have shown that 6PPD-Q decreases cell viability in rainbow trout gill cells (RTgill-W1)^42^ as well as in rat H9c2 cardiomyocytes^43^.

In the present study, we show that 6PPD, 6PPD-Q and DPPD exposure was associated with an increased level of DNA damage. Interestingly, a recent study by Wu et al. showed that 6PPD-Q can form DNA adducts in a concentration-dependent manner in A549 cells at the concentration range 2.7–13.4 µg/mL^28^. This type of DNA damage may be registered as strand breaks or alkali-labile sites in the comet assay, although it may also be a novel type of genotoxicity by a yet unresolved mechanism of action. Lockhart et al. found that 6PPD significantly reduced the levels of GSH in human myeloid HL-60 cells at a similar concentration range to our study^38^. Additionally, they found that pretreatment with NAC showed partial protection to 6PPD in regard to cell viability. This supports our findings that the addition of NAC protected against GSH depletion and DNA damage from 6PPD, which indicates ROS production as the primary cause of DNA damage. For 6PPD-Q and the other additives, the underlying mechanism is less certain. Since A549 cells possess some CYP-mediated metabolic activity^44^, it is possible that genotoxicity may also arise from electrophilic metabolites generated through metabolic activation. Fu et al. showed that 6PPD-Q induced senescence and DNA damage in Rat H9c2 cardiomyocytes. The former was found to be the result of ROS production and disruption of autophagy^43^.

*In* *vivo* studies support the above findings, showing that 6PPD-Q reached higher levels in the lungs compared to other organs tested in mice and induced inflammation as well as fibrosis^23^. 6PPD, 6PPD-Q and DPPD have also been found in other organs and induce toxicity or impair fertility in mice, rats and humans^22,45–47^. Interestingly, evidence from aquatic studies show sensitivity to 6PPD-Q may vary between similar species^27^. Epidemiological studies have linked 6PPD and 6PPD-Q in human samples to Parkinson’s disease^22^ and non-alcoholic fatty liver disease^21^. Lv et al. found that higher urinary excretion of 6PPD-Q was associated with increased risk of colorectal cancer^48^. Additionally, a study linked 6PPD-Q in dust with lower body mass index and higher incidence of influenza and diarrhoea in children^49^.

Previous studies highlight species- and organ-specific differences in the toxicity of 6PPD and 6PPD-Q, suggesting distinct mechanisms despite both being linked to acute cellular stress. A transcriptomic analysis of zebrafish liver tissue following a three-month *in vivo* exposure found distinct molecular responses to 6PPD and 6PPD-Q, although both induced hepatotoxicity. Specifically, 6PPD-Q had a greater impact on lipid biosynthesis and glycolysis inhibition^50^. This suggests different molecular responses exist to reach similar outcomes. 6PPD-Q and the other additives tested here may produce ROS at a different time-point or concentration not captured in our assay.

In summary, our finding that 6PPD, 6PPD-Q and DPPD can cause GSH-depletion and DNA damage in human lung cells is supported by the literature. There is evidence that the effects of 6PPD and 6PPD-Q may be species and organ specific.

### BTs

We find that BTZ and MBT affect cell cytotoxicity in either the WST-1 or LDH assay. The concentrations in the present study were too low to determine IC50, which is supported by the current literature. Ye et al. found concentration-dependent cytotoxic effects with BTZ and MBT in A549 and MGC-803 (human gastric carcinoma) cells, with IC50 values at higher concentrations than used in our study^51^. Supporting this, a study using mouse embryonic stem cells found an IC50 value of 169 µg/mL for BTZ^15^.

In our study, BTZ and MBT caused DNA damage in a concentration-dependent manner. To the best of our knowledge, there is only one other study on the comet assay where DNA strand break formation has been assessed after BTZ and MBT exposure^30^. The authors reported a statistically non-significant result for both BTZ and MBT with fish gill cells, although the reported data show a concentration-dependent increase in levels of DNA strand breaks by exposure to BTZ (calculated mean and standard deviation from three independent tests are 0.35 ± 0.04, 0.40 ± 0.2 and 0.60 ± 0.04 for 0, 75 and 150 µg/mL, which is statistically significant in ANOVA)^30^. In these salmon RTgill-W1 cells, BTZ and MBT were observed to increase ROS production after 2 h returning to a baseline level after 2 or 6 h, respectively^30^. The BTZ-derivative 2-hydroxybenzothiazole has been shown to increase ROS production, DNA strand breaks and apoptosis in mouse embryonic stem cells, as well as severely affecting growth and development of zebrafish embryos^15^. Noguerol et al. found that BTZ can bind and activate oestrogen receptors and aryl hydrocarbon receptors in recombinant yeast cells. This has the potential to contribute to DNA damage by ROS generation, cell cycle disruption and compromised DNA repair^52^.

In contrast to results on DNA strand breaks, studies on permanent manifestations of DNA damage have been largely negative. Studies on L5178Y mouse lymphoma cells (i.e. *thymidine kinase* mutations) and Chinese hamster lung V79 cells (i.e. *hypoxanthine–guanine phosphoribosyltransferase* mutations) showed unaltered mutagenicity after exposure to BTZ and MBT, respectively^53,54^. Studies from the National Toxicology Program indicated that MBT was mutagenic (L5178 mouse lymphoma cells) and clastogenic (chromosome aberrations and sister chromatid exchanges in Chinese hamster ovary cells) when exposed with metabolic activation^55^. Evidence exists that MBT can cause polyploidy, although not chromosomal aberrations, in Chinese hamster lung fibroblasts^29^. In addition, Ye et al. do not find BTZ or MBT to cause chromosomal-damaging effects with the micronucleus assay after 24 h exposure with both A549 and MGC-803 cells^51^. It should be noted that the report on micronuclei is not accompanied by results on proliferation index of the cells and there is no data on concurrent positive controls in the study. In general, the *in vitro* studies using cell lines from non-human species have shown mixed findings. Studies that have found no effect, have used mutation and chromosome damage assays to measure lesions that occur after cell division, which is different to immediate and potentially reversable DNA lesions that are detected in the comet assay.

Lastly, it should be noted that the International Agency for Research on Cancer (IARC) has evaluated MBT as probably carcinogenic to humans^56^. IARC highlights that there is epidemiological evidence where a positive association has been observed between exposure to MBT and cancer of the urinary bladder in workers at chemical production plants^1–5^. Additionally, there is sufficient evidence of carcinogenicity in experimental animals following exposure to MBT^55^. There is a paucity of observational and experimental studies on associations between BTZ exposure and carcinogenicity. Nevertheless, a recent epidemiological study looking at BTZ concentrations in urine samples finds strong evidence that BTZ is linked with lung cancer with participants in the highest quartile having a 95% higher risk of lung cancer^31^. *In vivo* studies have shown that inhalation of BTZ decreases the respiration rate of mice, suggesting it is an irritant^57^.

Our finding that BTZ and MBT cause GSH-depletion and DNA damage in human lung cells is supported by some literature; however, the overall evidence is mixed, with some studies reporting contradictory results.

## Conclusion

In conclusion, this study revealed that the tyre additives (6PPD, 6PPD-Q, DPPD, BTZ and MBT) caused GSH-depletion, as well as DNA damage in a concentration-dependent manner after 24 h exposure in A549 human lung epithelial cells. The inclusion of NAC at the time of exposure with 6PPD was able to mitigate these effects, suggesting that GSH-depletion contributed to the observed DNA damage. The concentrations of tyre additives, exposure durations and endpoints used in this study were chosen to assess acute effects and likely exceed those typically encountered by alveolar cells in real-world scenarios. However, the clear concentration-dependent responses observed in our *in vitro* model indicate a potential for genotoxicity. This is not sufficient to support comprehensive risk assessments but important for establishing an initial understanding of potential hazards to direct future studies. Several epidemiological studies reinforce concerns that prolonged low-dose exposure is associated with a range of adverse health effects. Given the environmental ubiquity of these additives, further research is needed to investigate toxicological effects at chronic low-dose concentrations and to uncover other mechanisms than oxidative stress by which they exert their effects. Specifically, long-term exposures may be more relevant to other DNA endpoints such as telomere length or persistent manifestations such as mutations, as well as for evaluating tumour development in a two-year bioassay as a hard outcome^58,59^.

## Data Availability

All data generated or analysed during this study are included in this published article. Raw data are available from the corresponding author upon request.
